# The chloroplast‐localized small heat shock protein Hsp21 associates with the thylakoid membranes in heat‐stressed plants

**DOI:** 10.1002/pro.3213

**Published:** 2017-06-26

**Authors:** Katja Bernfur, Gudrun Rutsdottir, Cecilia Emanuelsson

**Affiliations:** ^1^ Department of Biochemistry and Structural Biology Center for Molecular Protein Science, Lund University Sweden

**Keywords:** chaperone, heat shock protein, membranes, photosynthesis, quantitative mass spectrometry, stable isotope labeling, stress response, thermomemory

## Abstract

The small heat shock protein (sHsp) chaperones are crucial for cell survival and can prevent aggregation of client proteins that partially unfold under destabilizing conditions. Most investigations on the chaperone activity of sHsps are based on a limited set of thermosensitive model substrate client proteins since the endogenous targets are often not known. There is a high diversity among sHsps with a single conserved β‐sandwich fold domain defining the family, the α‐crystallin domain, whereas the N‐terminal and C‐terminal regions are highly variable in length and sequence among various sHsps and conserved only within orthologues. The endogenous targets are probably also varying among various sHsps, cellular compartments, cell type and organism. Here we have investigated Hsp21, a non‐metazoan sHsp expressed in the chloroplasts in green plants which experience huge environmental fluctuations not least in temperature. We describe how Hsp21 can also interact with the chloroplast thylakoid membranes, both when isolated thylakoid membranes are incubated with Hsp21 protein and when plants are heat‐stressed. The amount of Hsp21 associated with the thylakoid membranes was precisely determined by quantitative mass spectrometry after metabolic ^15^N‐isotope labeling of either recombinantly expressed and purified Hsp21 protein or intact *Arabidopsis thaliana* plants. We found that Hsp21 is among few proteins that become associated with the thylakoid membranes in heat‐stressed plants, and that approximately two thirds of the pool of chloroplast Hsp21 is affected. We conclude that for a complete picture of the role of sHsps in plant stress resistance also their association with the membranes should be considered.

**Abbreviations**
1‐DE: 1‐dimensional gel electrophoresis
CBB: Coomassie Brilliant Blue
DMSO: dimethylsulfoxide
EDTA: ethylene diamine tetraacetate
EGTA: ethylene glycol tetraacetic acid
FA: formic acid
LTQ: linear trap quadropole
MALDI: matrix‐assisted laser desorption ionization
MES: 2‐(N‐morpholino)ethanesulfonic acid
MOPS: 3‐(N‐morpholino)propanesulfonic acid
TFA: trifluoric acid.

## Introduction

The small heat shock proteins (sHsps) are oligomeric molecular chaperones that can interact with unfolding proteins and keep them in a transient state from which they can be refolded or degraded.^1^, ^2^, ^3^ The sHsps form a first line of defence against unfolding‐induced aggregation of cellular proteins. Whereas some sHsps become highly upregulated in response to stress other sHsps are constitutively expressed and important in the everyday life of cells. A number of severe human diseases are due to mutations in constitutively expressed sHsps, affecting cellular processes such as cell development, carcinogenesis, autophagy, apoptosis and the control of cytoskeletal architecture.^4^, ^5^ In plants the sHsps form an especially important part of the heat stress response.^6^, ^7^ The sHsp family is defined by the presence of a highly conserved α‐crystallin domain, which is flanked by N‐terminal and C‐terminal regions, which differ in length and sequence between different sHsps, and which control the assembly into oligomeric sHsp proteins with a dynamic subunit exchange.

The oligomeric sHsps form various complexes with model substrate client proteins,^8^, ^9^ with hundreds of variants of complexes observed in gas phase by nano‐electrospray mass spectrometry, with subunit exchange enhanced at higher temperature and further rearrangements in the quaternary structure. The mechanism and the molecular details of how sHsps interact with endogenous substrate client proteins, and how they operate to increase cellular stress resistance, is still not understood and most likely this is very different for different sHsps and organisms. The sHsps interactions with client proteins may be both transient and more stable, depending on the conditions.^10^ The sHsps may act as stability sensors through interaction with transiently populated unfolded states of proteins^11^ or react with unfolding proteins to form aggregates in which substrates are kept in near‐native states for refolding by ATP‐dependent chaperones in the proteostasis network.^12^, ^13^ In a series of recent papers highly interesting new possibilities are pointed out for the functioning of ATP‐independent chaperones, based on biophysical measurements on the *Escherichia coli* chaperone Spy,^14^, ^15^, ^16^ that was discovered in a genetic selection design to stabilize proteins. Four consecutive steps of chaperone‐client interactions without ATP are described, with folding of the client protein obtained while it was bound to the chaperones surface, and with the folding process itself sufficient to trigger the release.^17^

According to *in vitro* data on how sHsps prevent aggregation of thermosensitive model substrate client proteins the sHsps seem to operate at equimolar ratios.^1^, ^2^, ^3^ Therefore, it also remains enigmatic how the sHsps can interact with all the potential client proteins in the crowded environment in cells. Furthermore, in cells the proteins are not only dissolved in water, they are also surrounded by membranes. A phenomenon that is quite well described, yet often overlooked in attempts to understand the structure and function of sHsps, is that some sHsps have been reported to interact with and stabilize membranes, as reviewed in.^18^ A cyanobacterial sHsp, Hsp17, becomes associated with membranes under heat stress,^19^ and fluorescence anisotropy and FTIR measurements show that the association of Hsp17 decreases the fluidity of the membranes whereas a deletion *hsp17^‐^* mutational variant shows increased fluidity at high temperature.^20^ The sHsps may play a dual role in heat stress‐protection: as a chaperone in the soluble phase to protect the thermosensitive proteins that unfold and in the membranes to protect against and counteract the increased fluidity of the membranes.

Here, we address the question of whether Hsp21, a chloroplast‐localized sHsp in all photosynthesizing plants,^8^ also may associate with the thylakoid membranes of the chloroplast upon heat stress. A structural model of Hsp21 that we have obtained suggests a division of labor in the chaperone activity of Hsp21 such that the C‐terminal tails helps to maintain the oligomeric structure necessary for the chaperone activity, and the N‐terminal arms take part in the interactions with the substrate proteins.^21^ Plants have multiple sHsps with different intracellular location (in *Arabidopsis* >19 sHsps^22^), however Hsp21 is the only sHsp localized in the chloroplasts. The highest degree of sequence similarity to the chloroplast‐localized Hsp21 is found in the mitochondrial paralogue.^7^, ^23^ We have previously reported that Hsp21 overexpression increases plant stress resistance in *Arabidopsis thaliana*.^24^ Furthermore, Hsp21 was recently identified in an unbiased screen for factors responsible for thermomemory,^25^ the phenomenon that plants primed by a heat stress pretreatment can withstand subsequent heat stress better.

In this study, we have first used a controlled in vitro system, where recombinantly expressed and purified Hsp21‐protein have been incubated at different temperatures with thylakoid membranes isolated from *Arabidopsis thaliana* plants. The amount of Hsp21 associated to the thylakoid membranes upon heat stress was determined by quantitative mass spectrometry using 14N/^15^N isotope ratios. It was clearly shown that recombinantly expressed and purified Hsp21 protein associates with the thylakoid membrane after incubation at increased temperature. Furthermore, we have used an experimental system with intact *Arabidopsis* plants, and compared unlabeled (^14^N) control plants with isotope‐labeled (^15^N) heat stressed plants. By isolating chloroplasts from the two plant groups, and comparing the 14N/^15^N isotope ratios, on one hand in the thylakoid membranes from the two groups, and on the other hand in the soluble stroma from the two groups, we conclude that whereas 95% of the identified proteins were unaffected, remarkably two‐thirds of the pool of endogenously expressed Hsp21 became associated with the thylakoid membranes in the heat‐stressed plants.

## Results

### Quantification of recombinantly expressed and purified Hsp21 in thylakoid membranes

**Figure 1:**
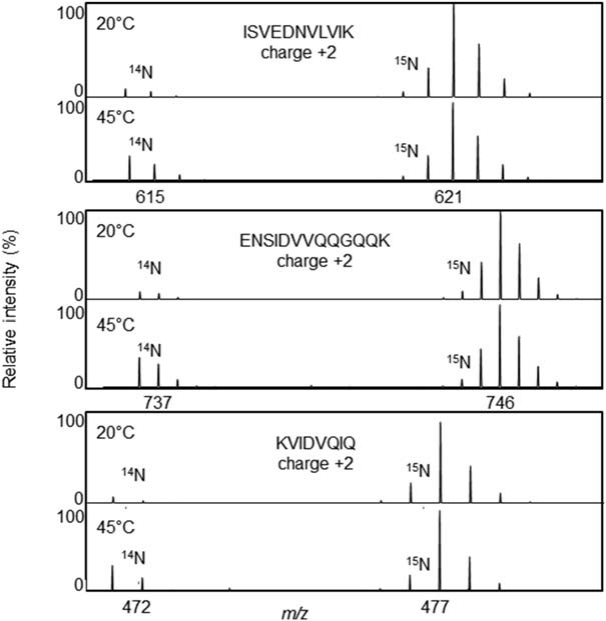
Hsp21 protein associates with thylakoid membranes at increased temperature. Mass spectra are shown for three different Hsp21 peptides (ISVEDNVLVIK, ENSIDVVQQGQQK, KVIDVQIQ, all charged 2+), showing an increased proportion of the 14N‐peaks at 45°C compared to 20°C, in relation to the reference 15N‐peaks. Isolated thylakoid membranes were incubated with recombinantly expressed and purified unlabeled (^14^N) Hsp21 for 15 min and samples spiked with known amounts of 15N‐labeled Hsp21 before sample processing, tryptic digestion and LC‐MSMS.

Protein labeling with stable isotopes like 15N is a powerful tool to use in mass spectrometric protein quantification and has been utilized in this study to quantify the amount of Hsp21 in various samples. The unlabeled (^14^N) and isotope‐labeled (^15^N) form of any one peptide will have identical ionization properties, which is the corner‐stone in quantitative mass spectrometry. The metabolic incorporation of 15N into proteins generates a mass increase in each peptide, proportional to the number of nitrogen atoms in that peptide. In tryptic digests of samples containing both the unlabeled (^14^N) and labeled (^15^N) form of a protein, each peptide will be visible as a peak pair in the mass spectra. Quantification is achieved by the Mascot Distiller Quantitation Toolbox software in the following way: each peptide is first identified with MS/MS, then the associated MS spectra are used to calculate the total intensity of the whole isotope cluster of the unlabeled (^14^N) peptide and the labeled (^15^N) peptide. The result is presented as *L*/*H*‐ratios, i.e. ratios between light 14N (L) and heavy 15N (H) peptide. An example of such peak pairs is shown in Figure 1 for three peptides ISVEDNVLVIK, ENSIDVVQQGQQK and APWDIKEEEHEIK. These data are derived from a sample where isolated thylakoid membranes were incubated at 20°C (upper panels) and 45°C (lower panels) in presence of unlabeled recombinantly expressed and purified Hsp21 protein, and subsequently spiked with known amount of 15N‐labeled Hsp21 protein as a reference. This figure shows that, with the peak intensity of the 15N‐labeled Hsp21 reference set to 100% in all panels, the peak intensity for the unlabeled Hsp21 (^14^N) is higher at 45°C compared to 20°C, for each of the three peptides and the amount Hsp21 associated with membrane therefore higher.

To explore how reproducible and reliable this quantification is, a set of samples were investigated with the results presented in Table 1. Three samples were prepared to be used as technical replicates (undiluted, diluted 1:10 and diluted 1:100) in which unlabeled (14N) and labeled (15N) Hsp21 were mixed 1:1 and subjected to LC‐MS/MS where in turn three different settings, on the mass spectrometer, for selecting peptides for MS/MS: mono isotopic selection on (MIPS on), mono isotopic selection off (MIPS off) and Inclusion list. The variations in *L*/*H*‐ratio between both the technical replicates and the different settings on the mass spectrometer were very small, suggesting that this is a robust protein quantification approach. The average *L*/*H*‐ratio is 1.22, thus although the samples were mixed at an intended 1:1 ration, it is clear that the absolute protein concentration in the 14N stock solution is approximately 20% higher than in the 15N protein stock solution. For the number of Hsp21 peptides selected for MS/MS there were small differences between the different settings; the number was higher if monoisotopic precursor selection was disenabled compared to the default setting (which is monoisotopic precursor selection enabled) and even higher if an inclusion list was used, which also best promoted fragmentation of 15N‐labeled peaks. This does not affect the *L*/*H*‐ratio, since the MS/MS‐data are only used for peptide identification and calculation of the *L/H*‐ratio is based on MS‐data. That the number of fragmented peptides is maximized in the acquisition mode with Inclusion list is however useful information for other applications which are based on MS/MS‐data.

**Table 1:** *Determination of* L/H*‐ratio in 1:1 Mixture of Recombinantly Expressed Hsp21 and ^15^N‐Labeled Hsp21*

	Acquisition mode^b^		
	MIPS off	MIPS on	Inclusion list
***L/H* ratio** a			
1 Undiluted	1.26	1.19	1.21
2 Diluted 1:10	1.22	1.28	1.21
3 Diluted 1:100	1.19	1.23	1.17
Average *L*/*H*	1.21	1.25	1.19

	L	H	%H	L	H	%H	L	H	%H
**Total # fragm peptides**
1 Undiluted	296	127	0,30	70	57	0,45	98	96	0,49
2 Diluted 1:10	65	31	0,32	48	26	0,35	281	255	0,48
3 Diluted 1:100	414	337	0,45	98	65	0,40	111	131	0,54
Average %H			0,36			0,40			0,50
**Unique # fragm peptides**
1 Undiluted	21	17	0,45	21	17	0,45	19	18	0,49
2 Diluted 1:10	10	5	0,33	6	4	0,40	39	36	0,48
3 Diluted 1:100	52	50	0,49	17	12	0,41	20	15	0,43
Average %H			0,42			0,42			0,47

a *L*/*H*‐ratio is the relative amount of unlabeled (^14^N) and ^15^N‐labeled Hsp21 determined as the ratio between the Light and the Heavy isotope with the Mascot Distiller Quantitation Toolbox in nine replicates with varying conditions in the experimental workflow and %H = H/(L + H). Unlabeled (^14^N) Hsp21 and ^15^N‐labeled Hsp21 at three different dilutions (undiluted, diluted 1:10, diluted 1:100) were mixed 1:1. The samples were run as references during electrophoresis, as described in Figure 2, and then subjected to tryptic in‐gel‐digestion. Three consecutive LC‐MSMS runs were made for each sample, with three different settings in the data acquisition mode with respect to the selection of precursor ions for MSMS fragmentation.

b Data acquisition modes were three different with respect to the selection of precursor ions for MSMS fragmentation: monoisotopic precursor selection enabled (MIPS on, which is the default setting) or disenabled (MIPS off) or with an inclusion list with theoretical masses for Hsp21 peptides (and MIPS off).

The data shown as an example in Figure 1 are fully presented in Figure 2. Samples of thylakoid membranes were incubated with Hsp21, as described above, separated by SDS‐PAGE as shown in Figure 2(A) and, as shown in Figure 2(B), the *L*/*H*‐ratios are approximately 0.25 and 0.05 for thylakoid membranes incubated at 45°C and 20°C, respectively. This corresponds to a five‐fold higher amount of Hsp21 in the thylakoid membranes at the increased temperature. The non‐dodecameric mutational variant, Hsp21^V181A^, was also detected in the thylakoid membranes after incubation at 45°C however also to some extent after incubation at 20°C. This is further commented in the Discussion.

**Figure 2:**
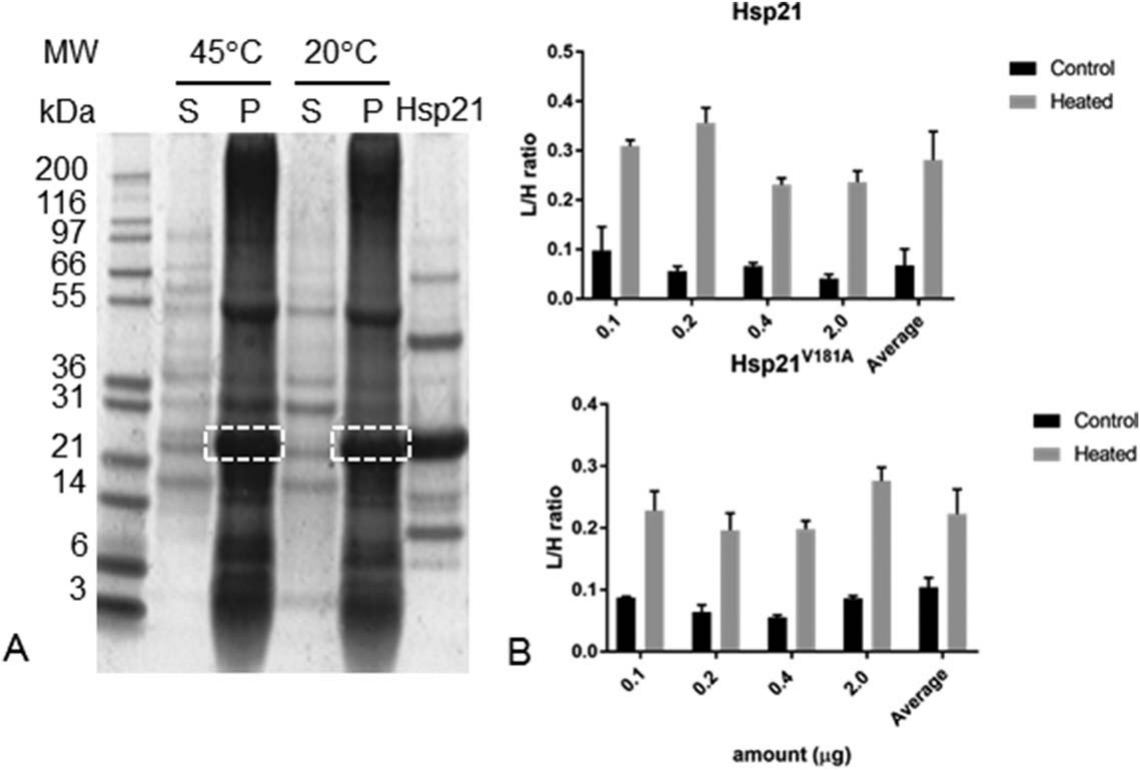
Quantification of Hsp21 protein associated with thylakoid membranes at increased temperature. A. Outline of the experimental set‐up in which thylakoid membranes were incubated at 20°C and 45°C with unlabeled (^14^N) Hsp21, and samples spiked with known amounts of 15N‐labeled Hsp21 before sample processing. By centrifugation pellet (P) with thylakoid membranes and supernatant (S) were separated, followed by SDS‐PAGE gfractionation. Samples excised (dashed rectangles) were subjected to LC‐MSMS to determine the *L*/*H*‐ratios. B. Triplicate samples of thylakoid membranes were processed as described in panel A and the relative amount of Hsp21 in thylakoid membranes incubated with Hsp21 at 20°C (Control) and 45°C (Heated) determined as *L*/*H*‐ratio. Results are shown for Hsp21 protein (upper panel) and for Hsp21^V181A^, a non‐dodecameric mutational variant (lower panel).

### Quantification of Hsp21 in thylakoid membranes in heat‐stressed plants

To investigate if Hsp21, when endogenously expressed in plant chloroplasts, may also associate with the thylakoid membranes, and to determine to what extent other proteins may also become associated, a proteomic work‐flow as outlined in Supporting Information Figure S1 was applied. Directly after heat stress, chloroplasts were rapidly isolated for analysis of their protein profiles. This was done by separating thylakoid membranes and soluble stroma, and then mix fractions of thylakoids and stroma, respectively, from heat‐stressed (^15^N‐labeled) and control plants (unlabeled, 14N) prior to LC‐MSMS and quantitative mass spectrometry. That there is no difference per se between plants metabolically labeled with 15N and unlabeled control plants is well known,^26^ thus the differences between heat‐stressed and control plants is therefore solely due to the increased temperature that the heat‐stressed (^15^N) plants were subjected to just before isolation of the thylakoid membranes.

**Figure 3:**
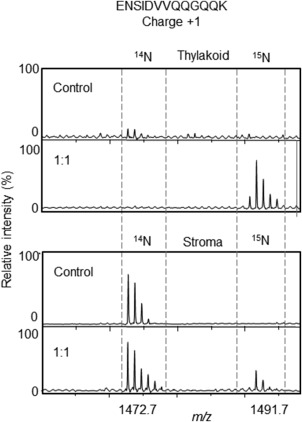
Hsp21 associates with thylakoid membranes in heat‐stressed plants. Mass spectra are shown for the Hsp21 peptide ENSIDVVQQGQQK detected in samples of Thylakoid membranes (upper), from control plants (^14^N) and from a 1:1 mix of control (^14^N) and heat‐stressed plants (^15^N), and samples of Stroma (lower), from control plants (^14^N) and from a 1:1 mix of control (^14^N) and heat‐stressed plants (^15^N). The Hsp21 peptide ENSIDVVQQGQQK is singly charged and has a mass difference of 19 Da between the 14N‐peptide (*m*/*z* = 1472.7) and the 15N‐peptide (*m*/*z* = 1491.7) due to 13 N‐atoms in each amido‐group, and 1 extra N‐atom in the amino acid side‐chains of N, Q and K. The quantification of the amount of Hsp21 in stroma and thylakoid membranes is shown as *L*/*H*‐ratios in Table 3.

The results are visualized in the mass spectra presented in Figure 3, showing the appearance of a 1:1 mixture of thylakoid membranes from control/heat‐stressed (^14^N/^15^N) plants in panel B with the 15N‐ peak of the Hsp21 peptide ENSIDVVQQGQQK very prominent (unlabeled control plants shown in panel A for comparison). The appearance is also shown of a 1:1 mixture of stroma fraction from control/heat‐stressed (^14^N/^15^N)‐plants in panel D with the 15N‐peak having low intensity compared to the corresponding 14N‐peak of the of the Hsp21 peptide ENSIDVVQQGQQK, (unlabeled control plants shown in panel C for comparison). These data show that there is Hsp21 in the thylakoid membranes of heat‐stressed (^15^N) plants, whereas no or very little in control (^14^N) plants. This provides evidence that Hsp21 associates with the thylakoid membranes not only in isolated thylakoid membranes incubated with recombinantly expressed and purified Hsp21 (Figs. 1 and 2), but also in heat‐stressed plants. Presence of Hsp21 in thylakoid membranes from heat‐stressed plants is also supported by an independent approach, immunoblotting (Supporting Information Fig. S2). It should be emphasized that this is the endogenously expressed Hsp21, which is 15N‐labeled as all the other proteins, in the 15N‐labeled *Arabidopsis thaliana* plants.

### Quantification of other proteins in thylakoid membranes in heat‐stressed plants

The proteomic data collected as outlined in Supporting Information Figure S1 could be used to quantify the relative amount of Hsp21 and other proteins in the thylakoid membranes in the heat‐stressed compared to control plants. The relative amount of Hsp21 in the thylakoid membranes was determined based on the 14N/^15^N peak pair intensities, as presented in Table 2. The *L*/*H*‐ratios for Hsp21 is very low, 0.05, which corresponds to a %*H* = *H*/(*H* + *L*) = 0.95. The other proteins detected in the same sample as Hsp21 show very much higher *L*/*H*‐ratios, ≫ 0.05. Many of these other proteins are subunits in the photosynthetic chlorophyll‐protein complexes which are very abundant thylakoid membrane proteins, with an average *L*/*H*‐ratio = 1.4 (%H = 0.42). This deviates from *L*/*H*‐ratio = 1 presumably due to a difference in the protein concentration determination of the stock solutions of the two thylakoid membrane preparations. To compensate the *L*/*H* ratios and %H‐values should be multiplied by 0.71 and 1.2, respectively, as also presented in separate columns in Table 2.

**Table 2:** Quantification of the Relative Amount of Various Proteins in the Thylakoid Membranes in Heat‐Stressed Plants Compared to Control Plants

Accession	Description	*L*/*H*	% *H* a	SD(geo)^b^	# pept^c^	Score^d^	Mass (kDa)	*L*/H*0.71^e^	%H*1.2^f^
AT4G27670.1	HSP21 heat shock protein 21	0.05	0.95	1.350	11	262	26	0.04	1.14
AT1G71500.1	Rieske (2Fe‐2S) domain‐containing protein	0.96	0.51	1.044	4	196	32	0.68	0.61
AT1G61520.1	#LHCA3 PS I light harvesting compl gene 3	1.08	0.48	1.071	8	554	29	0.77	0.58
AT5G66570.1	#PSBO‐1. OE33. PSBO1. MSP‐1 PS II oxygen‐evolving compl 1	1.10	0.48	1.144	3	132	36	0.78	0.57
AT4G10340.1	#LHCB5 light harvesting compl of PS II 5	1.11	0.47	1.055	18	1207	30	0.79	0.57
AT2G05070.1	#LHCB2.2. LHCB2 PS II light harvest compl gene 2.2	1.11	0.47	1.089	11	325	29	0.79	0.57
AT1G29910.1	#CAB3. AB180. LHCB1.2 chlorophyl A/B binding protein 3	1.13	0.47	1.095	9	287	28	0.81	0.56
AT2G34430.1	#LHB1B1. LHCB1.4 light‐harvest chl‐prot compl II subunit B1	1.14	0.47	1.085	9	304	28	0.81	0.56
AT3G23400.1	FIB4 Plastid‐lipid ass protein PAP/fibrillin family	1.14	0.47	1.033	3	170	30	0.81	0.56
AT1G44575.1	NPQ4. PSBS Chlorophyl A‐B binding family protein	1.18	0.46	1.059	7	374	28	0.83	0.55
AT5G54270.1	#LHCB3. LHCB3*1 light‐harvest chl B‐binding protein 3	1.21	0.45	1.071	5	109	29	0.86	0.54
AT4G02770.1	PSAD‐1 PS I subunit D‐1	1.22	0.45	1.089	6	323	23	0.86	0.54
AT1G54780.1	TLP18.3 thylakoid lumen 18.3 kDa protein	1.30	0.44	1.056	6	537	31	0.92	0.52
ATCG00020.1	#PSBA PS II reaction center protein A	1.43	0.41	1.044	9	472	39	1.02	0.49
AT1G06680.1	PSBP‐1. OEE2. PSII‐P. OE23 PS II subunit P‐1	1.47	0.41	1.043	7	646	28	1.04	0.49
AT4G21280.1	#PSBQ. PSBQA. PSBQ‐1 PS II subunit QA	1.48	0.40	1.128	4	132	24	1.05	0.48
AT4G03280.1	#PETC. PGR1 photosynthetic electron transfer C	1.54	0.39	1.082	3	146	24	1.09	0.47
AT3G61470.1	#LHCA2 PS I light harvesting compl gene 2	1.59	0.39	1.140	4	296	28	1.13	0.46
AT5G01530.1	#LHCB4.1 light harvesting compl PS II	1.61	0.38	1.495	4	274	31	1.14	0.46
AT1G15820.1	#LHCB6. CP24 light harvest compl PS II subunit 6	1.64	0.38	1.087	12	479	28	1.16	0.46
AT3G08940.2	#LHCB4.2 light harvesting compl PS II	1.73	0.37	1.453	4	103	31	1.23	0.44
AT3G26070.1	Plastid‐lipid ass protein PAP/fibrillin family	1.73	0.37	1.050	3	216	27	1.23	0.44
AT3G47470.1	#LHCA4. CAB4 light‐harvest chl‐prot compl I subunit A4	1.98	0.34	1.063	4	246	28	1.41	0.40

a The amount of protein in the thylakoid membrane in heat‐stressed plants compared to control plants is expressed as %H= H/L + H. based on the *L*/*H*‐ratio (Light (^14^N)/Heavy(^15^N)) calculated by the Mascot Distiller Toolbox software for at least 3 peptides as the sum of the signals from the heavy isotope divided by the sum of all signals of the light and heavy peptide.

b SD_geo_. parameter presented by the Mascot Distiller Quantitation Toolbox to estimate the average *L*/*H*‐ratios and should be >1.

c Number of unique peptides identified by MS/MS and used to determine *L*/*H*‐ratio.

d Mascot protein score.

Protein marked with # are 15 subunits in membrane‐bound chlorophyl‐protein complexes. with determined average *L*/*H*‐ratio = 1.4 and average %*H* = 0.42. Assuming that the abundance of these proteins do not change in the thylakoid membranes during a 2 h heat stress the values should be *L*/*H* = 1 and %*H* = 0.5. Thus multiplication with a factor 0.71 and 1.2, respectively, is required to reflect a situation with no changes in these 15 proteins.

Proteins detected with Mascot score > 100 and *L*/*H*‐ratio with SD (geo) > 1 in the gel segment at approximately 25 kDa excised after electrophoretic separation of proteins in a 1:1 mixture of thylakoid membranes from heat‐stressed (^15^N) plants and control (^14^N) plants

To assess how (un)common it is with such an increase in relative amount of a protein in the thylakoid membranes in heat‐stressed plants all segments of the electrophoresis gel were processed and analysed in twenty separate LC‐MSMS runs. For 104 proteins identified with a determined *L*/*H*‐ratio (Supporting Information Table S1), there were a few proteins with *L*/*H*‐ratio ≪ 1 and some with *L*/*H*‐ratio ≫1 but most of them were around *L*/*H* = 1 and none as low as Hsp21 with *L*/*H*‐ratio =0.05 (%*H* = 0.95). Thus with few exceptions most proteins did not associate the thylakoid membranes in the heat‐stressed plants, as Hsp21.

### Quantification of Hsp21 in the soluble stroma in heat‐stressed plants

**Figure 4:**
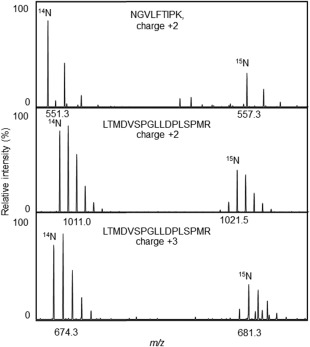
Decreased amount of Hsp21 in chloroplast stroma in heat‐stressed plants. Mass spectra are shown for three Hsp21 peptides (NGVLFTIPK charge 2+, LTMDVSPGLLDPLSPMR charge 2+, LTMDVSPGLLDPLSPMR charge 3+) detected in stroma samples with 1:1 mix of control (^14^N) and heat‐stressed plants (^15^N), showing a decreased proportion of the 15N‐peaks compared to 14N‐peaks indicating that the amount of Hsp21 in the soluble stroma is lower in the heat‐stressed compared to control plants. The quantification of the amount of Hsp21 in stroma and thylakoid membranes is shown as *L*/*H*‐ratios in Table III.

No or very little Hsp21 was detected in the thylakoid membranes of control plants, whereas a considerable amount of Hsp21 was detected in the thylakoid membranes from heat‐stressed plants (*L*/*H*‐ratio = 0.05, Table 2). To estimate how much of the pool of Hsp21 in the soluble stroma that becomes associated with the thylakoid membranes, the *L*/*H*‐ratio was determined for Hsp21 also in the soluble stroma fraction. Most of the soluble proteins in the chloroplast stroma do not change in abundance in heat‐stressed compared to control plants (Supporting Information Table S2). To specifically detect Hsp21 in the soluble stroma fraction, we subjected only the single excised gel segment corresponding to the position for Hsp21 [see Fig. 2(A)] to LC‐MSMS for a precise determination of the *L*/*H*‐ratio of Hsp21 in the soluble stroma fraction. As exemplified in the mass spectra for three peptides in Figure 4 and as shown in Table 3, the *L*/*H* ratio determined for Hsp21 in the soluble stroma is 2.34. This means that there is less Hsp21 in the soluble stroma in the heat‐stressed compared to control plants. The value for %H = H/(L + H) = 0.30 suggests that there is only a third of the pool of Hsp21 in the soluble stroma of the heat‐stressed plants. Thus the Hsp21 detected in the thylakoid membranes (Fig. 3, Table 2) should correspond to two thirds of the total amount of Hsp21 in the chloroplasts.

**Table 3:** Quantification of the Relative Amount of Hsp21 in the Chloroplast Soluble Stroma in Heat‐Stressed Plants Compared to Control Plants

z	Sequence	*L*/*H*	*L*/H*0.71	%*H*	%H*1.2	Std. Err.	Fraction	Correlation	Intensity
	**Stroma**								
2+	KVIDVQIQ	2.96	2.10	0.25	0.30	0.082	0.281	0.997	3 376
**2+**	**NGVLFITIPK**	**2.33**	**1.65**	**0.30**	**0.36**	**0.061**	**0.665**	**0.997**	**39 490**
2+	ISVEDNVLVIK	3.25	2.31	0.24	0.28	0.193	0.419	0.996	55 310
4+	APWDIKEEEHEIK	2.90	2.06	0.26	0.31	0.143	0.508	0.996	2 757
3+	APWDIKEEEHEIK	2.72	1.93	0.27	0.32	0.147	0.353	0.995	5 559
3+	ISVEDNVLVIKGEQK	2.43	1.72	0.29	0.35	0.136	0.181	0.996	14 130
**3+**	**LTMDVSPFGLLDPLSPMR**	**2.48**	**1.76**	**0.29**	**0.35**	**0.085**	**0.625**	**0.996**	**15 490**
**2+**	**LTMDVSPFGLLDPLSPMR**	**2.34**	**1.66**	**0.30**	**0.36**	**0.069**	**0.878**	**0.998**	**110 500**
**3+**	**QMLDTMDRMFEDTMPVSGR**	**2.20**	**1.56**	**0.31**	**0.37**	**0.040**	**0.790**	**0.996**	**17 190**
	**Average**	**2.34**							
	**Thylakoid membranes**								
2+	FDMPGLSK	0.17	0.12	0.85	1.02	2.219	0.413	0.953	458 000
2+	QMLDTMDR	0.03	0.02	0.97	1.17	0.028	0.247	0.980	6 954
**2+**	**MFEDTMPVSGR**	**0.03**	**0.02**	**0.97**	**1.17**	**0.002**	**0.204**	**0.989**	1 812 000
**2+**	**ENSIDVVQQGQQK**	**0.02**	**0.02**	**0.98**	**1.17**	**0.000**	**0.709**	**0.995**	1 269 000
2+	APWDIKEEEHEIK	0.05	0.04	0.95	1.14	0.081	0.061	0.997	61 230
4+	APWDIKEEEHEIK	0.02	0.01	0.98	1.18	0.005	0.243	0.996	61 840
3+	APWDIKEEEHEIK	0.02	0.01	0.98	1.18	0.089	0.068	0.969	180 500
**3+**	**LTMDVSPFGLLDPLSPMR**	**0.03**	**0.02**	**0.97**	**1.17**	**0.004**	**0.613**	**0.995**	972 200
**2+**	**LTMDVSPFGLLDPLSPMR**	**0.03**	**0.02**	**0.97**	**1.17**	**0.003**	**0.273**	**0.998**	3 780 000

The amount of protein in the thylakoid membrane in heat‐stressed plants compared to control plants is expressed as %H= H/L + H, based on the *L*/*H*‐ratio (Light (^14^N)/Heavy(^15^N)) calculated by the Mascot Distiller Toolbox software for at least 3 peptides as the sum of the signals from the heavy isotope divided by the sum of all signals of the light and heavy peptide.

SD_geo_, parameter presented by the Mascot Distiller Quantitation Toolbox to estimate the average *L*/*H*‐ratios and should be >1.

Number of unique peptides identified by MS/MS and used to determine *L*/*H*‐ratio.

Mascot protein score.

Peptides of the Hsp21 protein were detected in the gel segment at approximately 25 kDa excised after electrophoretic separation of proteins in a 1:1 mixture of soluble stroma fraction from heat‐stressed ^15^N‐plants and control ^14^N‐plants. As a comparison, corresponding data for chloroplast thylakoid membranes are also listed. Apart from the values for *L*/*H* and %*H*, determined as described in Table 2, four quality parameters (Standard error, Fraction, Correlation and Intensity) are presented which are used by the Mascot Distiller Quantitation Toolbox software to decide which peptides to include. The peptides that are included in the final calculation of average *L*/*H*‐ratio for the protein are indicated in bold.

## Discussion

Here we have shown, using stable isotope labeling and mass spectrometric determination of 14N/^15^N isotope ratios, data which suggest that Hsp21 associates with the thylakoid membrane at increased temperature, when isolated thylakoid membranes are incubated with Hsp21 protein (Figs. 1 and 2) and when *Arabidopsis thaliana* plants are heat‐stressed (Figs. 3 and 4, Tables 2 and 3). The increased amount of Hsp21 in the thylakoid membranes in the heat‐stressed compared to control plants is paralleled by a decreased amount of soluble Hsp21 in the chloroplast stroma, such that approximately two thirds of Hsp21 becomes associated with the thylakoid membranes (Table 3). Whereas this association with membranes is very pronounced for Hsp21, with few exceptions other proteins do not associate with the thylakoid membrane in heat‐stressed plants (Table 2, Supporting Information Table S1).

Both plant and yeast sHsps may aggregate at increased temperatures.^13^, ^27^ Whereas the possibility cannot be excluded that Hsp21 is detected in the thylakoid membranes merely due to aggregation in the pellet, we have previously documented the presence of Hsp21 in thylakoid membranes also when thylakoids are not isolated in a pellet but as a band at specific density in a sucrose density gradient.^28^ Aggregation appears to be very unlikely as an explanation also since numerous experiments with heating in the absence of the thylakoids show that Hsp21 is in fact very resistant to aggregation at 45°, e.g., in plate reader light‐scattering assays.^21^ Hsp21 stays in solution even when heated in presence of thermosensitive aggregation‐prone client proteins that largely are also maintained in solution (Supporting Information Fig. S3). Furthermore, the proteomics data from the in vivo experiments clearly show that hundreds of other proteins, that all should be expected to more aggregation‐prone than Hsp21, are not detected in the thylakoid membranes in response to the heat‐stress (Supporting Information Table S1).

That some sHsps may associate with membrane does not exclude that sHsps also target soluble substrate proteins, as suggested by their well‐documented ability to suppress the aggregation of thermosensitive model substrate proteins.^1^, ^3^ The case is open for sHsps to play more than one role and the chaperone activity assays with model substrates do not necessarily reflect the full functionality *in vivo*. For example, there are mutations in *Synechocystis* Hsp16.5 that impair its function of intact cells without a detectable effect on the chaperone activity as measured *in vitro*.^29^

That Hsp21 is crucial in thermomemory was discovered very recently, with data suggesting that Hsp21 and a plastid protease FtsH6 together form a control module for thermomemory in plants.^25^ Both Hsp21 and FtsH6 are strongly induced by heat stress, one after the other. The FtsH6 protease is responsible for Hsp21 degradation and the levels of Hsp21 remain high longer after heat stress in FtsH6 knock‐out plants, and in Hsp21 knock‐down plants the thermomemory is lost. The FtsH6 is a zink metalloprotease with a transmembrane domain, an AAA (ATPase associated with various cellular activities) domain and a protease domain facing the stroma, and thus stably inserted into the thylakoid membrane. It has previously been suggested to be involved in degradation of the membrane‐localized PSII light‐harvesting proteins.^30^ Since FtsH6 is localized in the thylakoid membrane, the translocation of Hsp21 into membrane would facilitate its degradation by FtsH6.

At present, it is not known in what form Hsp21 is inserted, or merely associated, with the thylakoid membrane. We have raised a non‐dodecameric mutational variant Hsp21^V181A^ to investigate the importance of the Hsp21 oligomerization.^21^ The substitution V181A in the IXI‐motif in the C‐terminal tail region destabilizes the dodecamer and Hsp21^V181A^ is composed of mainly dimers, in equilibrium with some hexamers. Here we noted that Hsp21^V181A^ was detected in the thylakoid membranes just as wildtype Hsp21 after incubation at increased temperature and also at non‐increased temperature, twice as much as the wildtype Hsp21 [Fig. 2(B)]. The hydrophobic surfaces, which are shielded in the dodecameric wildtype Hsp21, are presumably more exposed in the non‐dodecameric Hsp21^V181A^, which perhaps makes it more prone to interact with or penetrate into the membrane. It is generally assumed that the disordered and hydrophobic regions in sHsps are kept safely away in the oligomeric conformation, ready to be “unleashed” in order to perform the chaperoning activity upon oligomer disassembly.^1^, ^31^

One can speculate that the part being inserted into the membrane could be the amphipathic α‐helix motif in the N‐terminal domain of Hsp21.^24^, ^32^ Such an amphipathic α‐helix motif is commonly occurring in amphitrophic proteins which cycle between membrane phase and soluble phase in transduction of signals generated in cell membranes,^33^, ^34^ where the interaction with the membrane is often mediated by the amphipathic α‐helix.^35^ The N‐terminal domain in sHsps also displays properties typical for intrinsically disordered proteins^36^, ^37^, ^38^ and is presumably the substrate‐binding region that interacts with unfolding substrate proteins.^39^, ^40^ The amphipathic α‐helix motif in the N‐terminal domain of Hsp21 contains highly conserved methionines.^32^, ^41^ We have previously shown that these methionines can undergo reversible methionine sulfoxidation due to the presence of a chloroplast‐specific form of methionine sulfoxide reductase,^42^ suggesting an Hsp21 methionine sulfoxidation‐reduction cycle to quench reactive oxygen species.^28^ Thus the association of Hsp21 with the thylakoid membranes could also act to protect the membrane against oxygen radicals that cause lipid peroxidation, as shown for the sHsp in *Mycobacterium tuberculosis*.^43^

It remains to be investigated whether dissociated subunits of Hsp21 (monomeric or dimeric subunits) interact with the membrane and whether Hsp21 is just inserted into the lipid bilayer or interacting with soluble parts of integral membrane proteins that may unfold in response to increased temperature. That Hsp21 may directly interact with and stabilize the membrane‐bound Photosystem II core subunits has been suggested.^44^ There are a number of publications pointing out the possible importance of the interaction of the cyanobacterial Hsp17 with the membrane lipids,^19^, ^20^, ^45^ for regulation of the membrane fluidity and preservation of the membrane integrity during thermal fluctuations. Also, higher plants have to cope with extreme variations in temperature, so control of the membrane fluidity may be as relevant for plant membranes as for cyanobacterial membranes. It also remains to be investigated whether the cytosolic sHsps^46^ can associate with the plasma membrane and/or the chloroplast envelope membrane.

To summarize, some sHsps may be partially present in membranes, as a membrane sHsp subfraction, which does not exclude that the soluble sHsp subfraction also fulfills the role to bind destabilized proteins in their more hydrophobic partially or fully unfolded forms. Our data further emphasize that attempts to understand the physiological role of Hsp21 in plant stress resistance should take into account its possible association with membranes.

## Materials and Methods

### Expression and purification of Hsp21 protein without and with 15N‐labeling

Recombinantly expressed Hsp21 from *Arabidopsis thaliana* (sequence as in UniProtKB P31170 with a start methionine replacing the first 44 amino acids, which are the transit sequence not part of the mature protein within the chloroplast^47^), and non‐dodecameric mutational variant Hsp21^V81A^, were expressed and purified as previously described.^21^ Hsp21 protein labeled with 15N was obtained by growing the bacterial host in minimal medium containing 15N‐NH_4_Cl as nitrogen source and was purified as the unlabeled protein. The proteins were finally collected in 50 mM HEPES pH 8, 100 mM NaCl, 10 mM DTT, 5 mM MgCl_2_. Protein concentration of the stock solutions of Hsp21 and 15N‐labeled Hsp21 was determined by triplicate measurements of the absorbance at 280 nm using a NanoDrop 2000 spectrophotometer (Thermo‐Fisher Scientific, Wilmington, DE, USA), and stored in aliquots at −20°C.

### Heat‐treatment of isolated thylakoid membranes in presence of purified Hsp21 protein

Thylakoid membranes, that had been isolated from *Arabidopsis thaliana* plants, concentration‐determined and stored in aliquots at −80°C as described in,^26^ were thawed immediately before use. Stock solutions of Hsp21 and thylakoid membranes were diluted 10 or 100 times with buffer (20 mM sodium phosphate buffer pH 8, 150 mM NaCl) to prepare samples with various amounts of Hsp21 (2, 0.4, 0.2, and 0.1 µg) and thylakoid membranes corresponding to 75 µg protein in a total volume of 24 µl. After incubation at 20 or 45°C for 15 min the thylakoid membranes were isolated and washed in the following way: after centrifugation (Eppendorf centrifuge, 5 min 4000 rpm), 12 µl (half of each sample) was withdrawn from the top of the supernatant and spiked with an amount of 15N‐labeled Hsp21 corresponding to half of the originally added amount of unlabeled Hsp21. The rest of the supernatant was discarded and the pellet with the thylakoid membranes was washed with 24 µl fresh buffer. After centrifugation (Eppendorf centrifuge, 5 min 4000 rpm), the washed pellet was dissolved in 12 µl fresh buffer and spiked with an amount of 15N‐labeled Hsp21 corresponding to the amount of unlabeled Hsp21 originally added. Loading buffer with LDS and DTT were added and samples heated for 10 min at 95°C before loading for SDS‐PAGE.

### Heat‐treatment of plants containing endogenously expressed Hsp21 and subsequent isolation of thylakoid membranes

Plants of *Arabidopsis thaliana* ecotype Columbia (Col‐0) were grown hydroponically, either without or with stable isotope labeling (^14^N‐plants and 15N‐plants, respectively) as described in.^26^ Heat‐treatment was performed in a VB0714 Hereaus‐Vötsch climate‐chamber (Hereaus, Mölndal, Sweden) with humidity kept at 70% to prevent transpiration. To induce expression of Hsp21 all plants were subjected to a heat pretreatment on day 46 (45°C for 30 min, and the next day 45°C for 90 min). After another three days, the 15N‐plants were subjected to heat‐treatment (45°C for 60 min) whereas the 14N plants were kept as control plants in the growth chamber. All plants were then immediately transferred to the cold room and chloroplast isolation was performed at 4°C with all materials and buffers precooled. Separation of the soluble stroma and the thylakoid membrane fraction was performed as described in.^26^ The protein concentrations were determined and fractions stored in aliquots at −80°C. To quantify the amount of Hsp21 in heat‐stressed versus control membranes, and heat‐stressed versus control stroma, fractions from heat‐stressed 15N‐plants and control 14N‐plants were thawed, and mixed 1:1 as described in^26^ and loaded for SDS‐PAGE.

### Denaturing electrophoresis and tryptic in‐gel‐digestion

After SDS‐PAGE (denaturing electrophoresis) samples were processed further to perform mass spectrometric quantification of the amount of Hsp21, by excision of gel bands corresponding to approximately 20 kDa, guided by the position of a reference sample Hsp21, and tryptic in‐gel‐digestion. In‐gel digestion was performed after de‐staining 1x1 mm gel pieces in 50 mM ammonium bicarbonate/50% ACN until colorless, washing in double‐distilled water and dehydration in 100% ACN. Subsequently digestion buffer was added (10 mM ammonium bicarbonate/10% ACN with 12 ng/µl sequencing grade modified trypsin (Promega Biotech AB, Nacka, Sweden)) and incubated on ice for 2 h. After further addition of 50 mM ammonium bicarbonate samples were incubated at 37°C overnight. The overnight digestion solutions were saved and pooled with an aliquot of further extraction from gel pieces in extraction buffer (1:2 (vol/vol) 5% formic acid/ACN) for 30 min at 37°C.

### Mass spectrometry

The tryptic digests were first inspected by acquisition of MALDI‐MS spectra using a 4700 Proteomics Analyzer (Applied Biosystems/MDS SCIEX, USA) in the positive reflector mode. Peptides were then subjected to reversed phase nano‐LC coupled to an LTQ‐Orbitrap Velos Pro mass spectrometer (Thermo Fisher Scientific, Stockholm, Sweden) equipped with a nano Easy spray ion source (Proxeon Biosystems, Odense, Denmark). The chromatographic separation was performed at 40°C on a 15 cm (75 μm i.d.) EASY‐Spray column packed with 3 μm resin The nano HPLC intelligent flow control gradient was created by solvent A (0.1% (v/v) FA in water) and solvent B (0.1% (v/v) FA in 100% (v/v) acetonitrile) and the gradient was run as follows: 5–30% during 40 min, 30–50% during 20 min and 50–95% during 5 min and constant at 95% for 10 min. A flow rate of 300 nl/min was used through the gradient. An MS scan (400–1400 *m*/*z*) was recorded in the Orbitrap mass analyzer set at a resolution of 60,000 at 400 *m*/*z*, 1 × 106 automatic gain control target and 500 ms maximum ion injection time. The MS was followed by data‐dependent collision‐induced dissociation MS/MS scans on the 4 most intense ions in the LTQ at 2500 signal threshold, 30,000 automatic gain control target, 300‐ms maximum ion injection time, 3.0 *m*/*z* isolation width, 10 ms activation time at 35 normalized collision energy and dynamic exclusion enabled for 30 or 600 s with a repeat count of 2, auxiliary gas flow; S‐lens 60%; ion transfer tube temperature, 275°C.

### Processing of mass spectrometric data

With an in‐house licensed version of the software Mascot Server software (version 2.5, Matrix Science Inc, Boston, USA, http://www.matrixscience.com) raw files, typically containing 20,000 scans each, were converted to mgf‐format by Mascot Distiller (version 2.6) and identification of proteins were performed with the Mascot Daemon software (version 2.4). For the conversion of the raw‐files from the Thermo Xcalibur software the default settings were optimized, in MS and MSMS processing: Peak half width 0.02, 600 data points per Da, in Time domain Max intermediate time 0, in MSMS and MS peak picking same settings with Min S/N =2, Peak profile Min peak width 0.002, Expected 0.02, Max 0.2. For protein identification the following parameter settings were used: UniProt database, subsection SwissProt restricted to *Arabidopsis thaliana*, Enzyme: trypsin, missed cleavage sites: 1 or 2, variable modification: methionine oxidation, peptide mass tolerance: 10 ppm, fragment ion mass tolerance: 0.05 Da, Quantitation: 15N Metabolic. The Mascot Distiller Quantitation Toolbox was used to assess the *L*/*H* ratio between light (^14^N) and heavy (^15^N) isotope. The protocol requires information from the raw data file that is not present in the peak list, and Mascot Distiller has access to both the Mascot search results and the raw data. The mass spectrometry raw data are available at ProteomeXchange (http://proteomexchange.org/) with the dataset identifier PXD006734.

## Conflict of Interest Statement

The authors declare no competing interests to disclose.

## Supporting information

Supporting Information Figure 1.

Supporting Information Figure 2.

Supporting Information Figure 3.

Supporting Information Table 1.

Supporting Information Table 2.
