# Supplementary figures and images for: The chloroplast‐localized small heat shock protein Hsp21 associates with the thylakoid membranes in heat‐stressed plants
Source: Protein Sci. 2017 Jun 26;26(9):1773–84. doi: 10.1002/pro.3213 (PMC5563132; doi:10.1002/pro.3213)

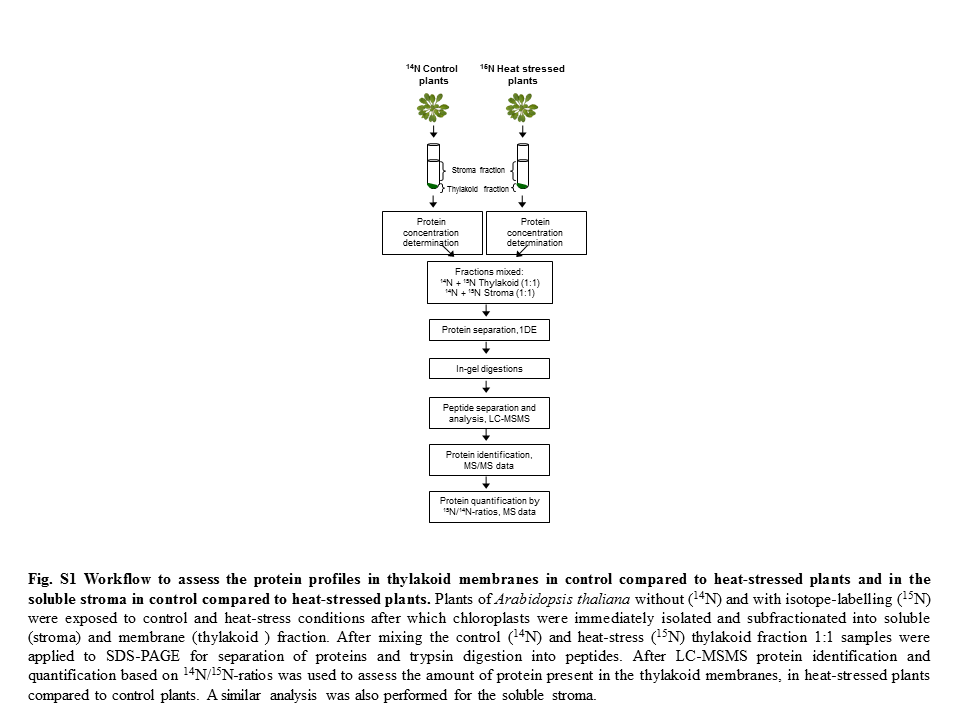

Supplement: Supplementary file 1 — Supporting Information Figure 1. [file PRO-26-1773-s001.TIF]

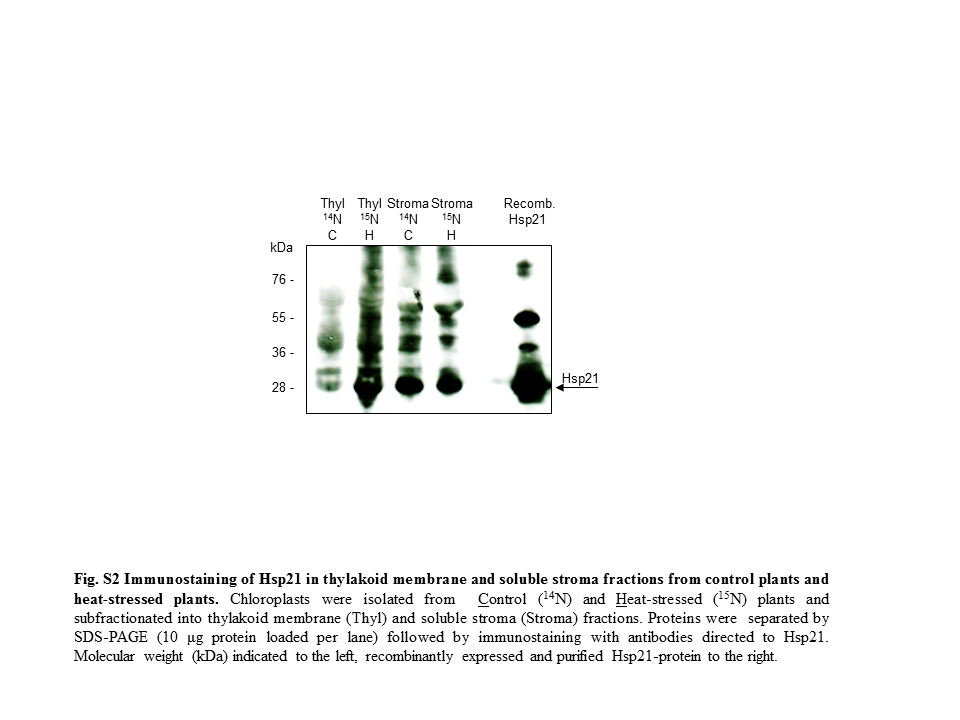

Supplement: Supplementary file 2 — Supporting Information Figure 2. [file PRO-26-1773-s002.TIF]

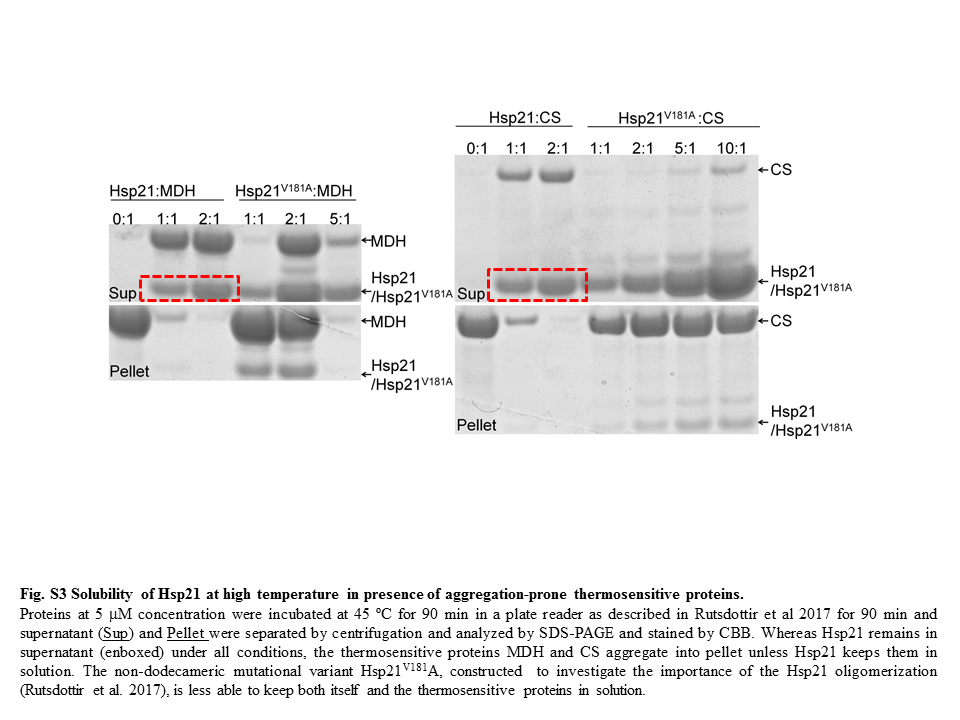

Supplement: Supplementary file 3 — Supporting Information Figure 3. [file PRO-26-1773-s003.TIF]
