# Supplementary material for: The chloroplast‐localized small heat shock protein Hsp21 associates with the thylakoid membranes in heat‐stressed plants
Source: Protein Sci. 2017 Jun 26;26(9):1773–84. doi: 10.1002/pro.3213 (PMC5563132; doi:10.1002/pro.3213)
Supplement: Supplementary file 4 — Supporting Information Table 1. [file PRO-26-1773-s004.docx]

**Table S1. Quantification of the relative amount of proteins in the thylakoid membranes from heat-stressed compared to control plants.** Proteins were detected in a 1:1 mixture of thylakoid membrane fraction from heat-stressed ^15^N-plants and control ^14^N-plants and the L/H ratio determined as outlined in Fig. S1. Protein identification was obtained for 970 proteins, of which 104 proteins obtained a score > 100 and a determined L/H ratio with SD_geo_ >1 in Mascot Distiller Quantitation toolbox. From the L/H-ratio is calculated %H = H/(L+H). Based on the assumption made in Table 3 that the membrane protein subunits in the chlorophyll-protein complex of Photosystem 2 do not change in abundance during 2 h heat stress the determined L/H-ratios and %H-values are multiplied by a factor 0.71 and 1.2, respectively, to reflect a situation with no changes in the 15 proteins. Mass spectrometry raw data related are made available publically at ProteomeXchange (http://www.proteomexchange.org/).

Samples from approximately 20 excised bands were subjected to LC-MSMS. The data set contains, for each of heat-stressed and control plants, 20 biological replicates (n=20), which were pooled in order to minimize the biological variation. Several technical replicates were also obtained, since the same protein was often detected in in more than one of the excised gel segments (in 50% of the proteins L/H-ratio determined in >1 segment, for 20% in > 3 segments).

| **Accession** | **Description** | **L/H** | **%H** | **SD(geo)** | **# pept** | **Score** | **Mass (Da)** | **L/H * 0.71** | **%H***  **1.2** |
| --- | --- | --- | --- | --- | --- | --- | --- | --- | --- |
|  |  |  |  |  |  |  |  |  |  |
| AT4G27670.1 | HSP21 heat shock protein 21 | 0.05 | 0.95 | 1.35 | 11 | 262 | 25644 | 0.04 | 1.14 |
| AT4G39730.1 | Lipase/lipooxygenase, PLAT/LH2 family protein | 0.24 | 0.81 | 1.79 | 3 | 122 | 20408 | 0.17 | 0.97 |
| AT5G09660.2 | PMDH2 peroxisomal NAD-malate dehydrogenase 2 | 0.33 | 0.75 | 1.04 | 3 | 281 | 35296 | 0.24 | 0.90 |
| AT5G49910.1 | CPHSC70-HEAT SHOCK PROTEIN 70-2, HSC70-7, cpHsc70-2 | 0.37 | 0.73 | 1.07 | 2 | 468 | 77064 | 0.26 | 0.88 |
| AT2G35410.1 | RNA-binding (RRM/RBD/RNP motifs) family protein | 0.44 | 0.70 | 1.10 | 3 | 345 | 34294 | 0.31 | 0.83 |
| AT4G37930.1 | SHM1, STM, SHMT1 serine transhydroxymethyltransferase 1 | 0.47 | 0.68 | 1.23 | 2 | 167 | 58229 | 0.33 | 0.82 |
| AT3G12580.1 | HSP70, ATHSP70 heat shock protein 70 | 0.47 | 0.68 | 1.02 | 3 | 507 | 72324 | 0.33 | 0.82 |
| AT3G09440.1 | Heat shock protein 70 (Hsp 70) family protein | 0.47 | 0.68 | 1.02 | 4 | 358 | 71559 | 0.33 | 0.82 |
| AT5G01920.1 | STN8 Protein kinase superfamily protein | 0.54 | 0.65 | 6.49 | 3 | 155 | 55229 | 0.38 | 0.78 |
| AT3G13470.1 | TCP-1/cpn60 chaperonin family protein | 0.57 | 0.64 | 1.04 | 3 | 443 | 64475 | 0.41 | 0.76 |
| AT1G16720.1 | HCF173 high chlorophyll fluorescence phenotype 173 | 0.62 | 0.62 | 1.11 | 7 | 359 | 66954 | 0.44 | 0.74 |
| AT3G25860.1 | LTA2, PLE2 2-oxoacid dehydrogenases acyltransferase family protein | 0.65 | 0.61 | 1.29 | 4 | 428 | 50106 | 0.46 | 0.73 |
| AT4G20360.1 | ATRAB8D, ATRABE1B, RABE1b RAB GTPase homolog E1B | 0.67 | 0.60 | 1.05 | 2 | 112 | 51883 | 0.48 | 0.72 |
| ATCG00490.1 | RBCL ribulose-bisphosphate carboxylases | 0.68 | 0.60 | 1.02 | 3 | 129 | 53435 | 0.48 | 0.72 |
| AT2G39730.1 | RCA rubisco activase | 0.75 | 0.57 | 1.22 | 2 | 106 | 52984 | 0.53 | 0.69 |
| AT5G51545.1 | LPA2 low psii accumulation2 | 0.81 | 0.55 | 1.05 | 3 | 171 | 20314 | 0.57 | 0.66 |
| AT3G09580.1 | FAD/NAD(P)-binding oxidoreductase family protein | 0.81 | 0.55 | 1.16 | 4 | 297 | 52397 | 0.57 | 0.66 |
| AT2G42130.2 | Plastid-lipid associated protein PAP / fibrillin family protein | 0.83 | 0.55 | 1.17 | 2 | 245 | 29821 | 0.59 | 0.66 |
| AT5G50920.1 | CLPC, ATHSP93-V, HSP93-V, DCA1, CLPC1 CLPC homologue 1 | 0.84 | 0.54 | 1.08 | 3 | 718 | 104922 | 0.59 | 0.65 |
| AT3G51820.1 | ATG4, G4, CHLG UbiA prenyltransferase family protein | 0.86 | 0.54 | 1.11 | 2 | 242 | 42083 | 0.61 | 0.64 |
| AT1G80030.1 | Molecular chaperone Hsp40/DnaJ family protein | 0.90 | 0.53 | 1.10 | 5 | 279 | 54300 | 0.64 | 0.63 |
| AT5G26742.1 | emb1138 DEAD box RNA helicase (RH3) | 0.93 | 0.52 | 1.13 | 3 | 398 | 82323 | 0.66 | 0.62 |
| AT4G19100.1 | PAM68 Protein of unknown function (DUF3464) | 0.98 | 0.51 | 1.03 | 2 | 120 | 24367 | 0.69 | 0.61 |
| ATCG00150.1 | ATPI ATPase, F0 complex, subunit A protein | 0.99 | 0.50 | 1.14 | 2 | 104 | 27688 | 0.70 | 0.60 |
| AT5G07020.1 | Proline-rich family protein | 1.03 | 0.49 | 1.07 | 2 | 334 | 24775 | 0.73 | 0.59 |
| AT1G57720.1 | Translation elongation factor EF1B, gamma chain | 1.05 | 0.49 | 1.11 | 3 | 213 | 46598 | 0.74 | 0.59 |
| AT1G09640.1 | Translation elongation factor EF1B, gamma chain | 1.05 | 0.49 | 1.11 | 3 | 208 | 46859 | 0.74 | 0.59 |
| AT4G09010.1 | APX4, TL29 ascorbate peroxidase 4 | 1.07 | 0.48 | 1.01 | 7 | 651 | 38082 | 0.76 | 0.58 |
| AT4G32770.1 | VTE1, ATSDX1 tocopherol cyclase/ sucrose export defective 1 (SXD1) | 1.10 | 0.48 | 1.19 | 2 | 465 | 55142 | 0.78 | 0.57 |
| AT5G66570.1 | PSBO-1, OE33, PSBO1, MSP-1 PS II oxygen-evolving complex 1 | 1.10 | 0.48 | 1.14 | 3 | 132 | 35528 | 0.78 | 0.57 |
| AT4G22240.1 | Plastid-lipid associated protein PAP / fibrillin family protein | 1.12 | 0.47 | 1.09 | 4 | 374 | 33692 | 0.80 | 0.57 |
| ATCG01010.1 | NDHF NADH-Ubiquinone oxidoreductase (complex I), chain 5 protein | 1.13 | 0.47 | 1.02 | 3 | 166 | 85696 | 0.80 | 0.56 |
| AT1G61520.1 | LHCA3 PS I light harvesting complex gene 3 | 1.15 | 0.47 | 1.08 | 5 | 115 | 29163 | 0.82 | 0.56 |
| ATCG01110.1 | NDHH NAD(P)H dehydrogenase subunit H | 1.17 | 0.46 | 1.10 | 2 | 153 | 45644 | 0.83 | 0.55 |
| AT1G03130.1 | PSAD-2 PS I subunit D-2 | 1.18 | 0.46 | 1.18 | 16 | 1766 | 22623 | 0.83 | 0.55 |
| AT3G08920.1 | Rhodanese/Cell cycle control phosphatase superfamily protein | 1.18 | 0.46 | 1.09 | 2 | 113 | 23822 | 0.84 | 0.55 |
| AT1G54500.1 | Rubredoxin-like superfamily protein | 1.19 | 0.46 | 1.07 | 5 | 289 | 22146 | 0.84 | 0.55 |
| AT2G20260.1 | PSAE-2 PS I subunit E-2 | 1.20 | 0.45 | 1.08 | 5 | 184 | 15180 | 0.85 | 0.54 |
| AT5G54270.1 | LHCB3, LHCB3*1 light-harvesting chlorophyll B-binding protein 3 | 1.21 | 0.45 | 1.07 | 5 | 109 | 28688 | 0.86 | 0.54 |
| AT4G35250.1 | NAD(P)-binding Rossmann-fold superfamily protein | 1.22 | 0.45 | 1.25 | 3 | 106 | 44152 | 0.86 | 0.54 |
| AT5G61790.1 | CNX1, ATCNX1 calnexin 1 | 1.22 | 0.45 | 1.21 | 3 | 120 | 60790 | 0.86 | 0.54 |
| AT2G34460.1 | NAD(P)-binding Rossmann-fold superfamily protein | 1.22 | 0.45 | 1.06 | 16 | 812 | 30566 | 0.87 | 0.54 |
| AT3G47860.1 | CHL chloroplastic lipocalin | 1.24 | 0.45 | 1.05 | 2 | 183 | 39775 | 0.88 | 0.53 |
| AT1G02910.1 | LPA1 tetratricopeptide repeat (TPR)-containing protein | 1.25 | 0.44 | 1.13 | 2 | 315 | 50936 | 0.89 | 0.53 |
| AT2G42220.1 | Rhodanese/Cell cycle control phosphatase superfamily protein | 1.27 | 0.44 | 1.11 | 3 | 181 | 25722 | 0.90 | 0.53 |
| AT4G02770.1 | PSAD-1 PS I subunit D-1 | 1.29 | 0.44 | 1.13 | 5 | 131 | 22641 | 0.91 | 0.52 |
| AT1G50250.1 | FTSH1 FTSH protease 1 | 1.29 | 0.44 | 1.13 | 6 | 405 | 77823 | 0.91 | 0.52 |
| AT1G51110.1 | Plastid-lipid associated protein PAP / fibrillin family protein | 1.29 | 0.44 | 1.20 | 4 | 201 | 46079 | 0.92 | 0.52 |
| AT3G26060.1 | ATPRX Q Thioredoxin superfamily protein | 1.30 | 0.44 | 1.07 | 5 | 197 | 24117 | 0.92 | 0.52 |
| AT2G07698.1 | ATPase, F1 complex, alpha subunit protein | 1.34 | 0.43 | 1.27 | 3 | 246 | 86221 | 0.95 | 0.51 |
| AT1G56500.1 | Haloacid dehalogenase-like hydrolase family protein | 1.35 | 0.43 | 1.01 | 2 | 252 | 115072 | 0.96 | 0.51 |
| AT5G13120.1 | ATCYP20-2, CYP20-2 cyclophilin 20-2 | 1.35 | 0.42 | 1.20 | 2 | 129 | 28516 | 0.96 | 0.51 |
| AT1G15980.1 | NDF1, NDH48 NDH-dependent cyclic electron flow 1 | 1.37 | 0.42 | 1.10 | 10 | 654 | 51275 | 0.97 | 0.51 |
| AT3G61870.1 | Unknown protein; FUNCTIONS IN: molecular_function unknown | 1.39 | 0.42 | 1.10 | 5 | 403 | 29681 | 0.98 | 0.50 |
| AT5G17170.1 | ENH1 rubredoxin family protein | 1.40 | 0.42 | 1.11 | 10 | 1030 | 28669 | 0.99 | 0.50 |
| AT5G21430.1 | Chaperone DnaJ-domain superfamily protein | 1.41 | 0.42 | 1.06 | 6 | 688 | 24593 | 1.00 | 0.50 |
| AT3G26080.1 | Plastid-lipid associated protein PAP / fibrillin family protein | 1.44 | 0.41 | 1.02 | 2 | 239 | 26346 | 1.02 | 0.49 |
| ATCG01060.1 | PSAC iron-sulfur cluster binding;electron carriers | 1.44 | 0.41 | 1.03 | 2 | 139 | 9545 | 1.02 | 0.49 |
| AT3G08580.1 | AAC1 ADP/ATP carrier 1 | 1.46 | 0.41 | 1.10 | 4 | 239 | 41563 | 1.04 | 0.49 |
| AT4G27700.1 | Rhodanese/Cell cycle control phosphatase superfamily protein | 1.48 | 0.40 | 1.32 | 3 | 262 | 24971 | 1.05 | 0.48 |
| AT3G61470.1 | LHCA2 PS I light harvesting complex gene 2 | 1.59 | 0.39 | 1.14 | 4 | 296 | 27737 | 1.13 | 0.46 |
| AT1G79600.1 | Protein kinase superfamily protein | 1.60 | 0.38 | 1.12 | 3 | 295 | 79201 | 1.14 | 0.46 |
| AT4G32260.1 | ATPase, F0 complex, subunit B/B', bacterial/chloroplast | 1.62 | 0.38 | 1.17 | 5 | 229 | 23903 | 1.15 | 0.46 |
| AT5G64040.1 | PSAN PS I reaction center subunit PSI-N, chloroplast, putative / PSI-N | 1.62 | 0.38 | 1.00 | 2 | 107 | 18702 | 1.15 | 0.46 |
| AT1G34000.1 | OHP2 one-helix protein 2 | 1.63 | 0.38 | 1.10 | 2 | 132 | 18825 | 1.15 | 0.46 |
| AT2G33800.1 | Ribosomal protein S5 family protein | 1.64 | 0.38 | 1.67 | 3 | 167 | 32682 | 1.17 | 0.45 |
| AT5G23120.1 | HCF136 PS II stability/assembly factor | 1.65 | 0.38 | 1.03 | 2 | 256 | 44133 | 1.17 | 0.45 |
| AT5G26000.2 | TGG1, BGLU38 thioglucoside glucohydrolase 1 | 1.66 | 0.38 | 1.04 | 4 | 307 | 51961 | 1.18 | 0.45 |
| AT5G35630.1 | GS2, GLN2, ATGSL1 glutamine synthetase 2 | 1.68 | 0.37 | 1.07 | 2 | 165 | 47780 | 1.19 | 0.45 |
| ATCG00020.1 | PSBA PS II reaction center protein A | 1.69 | 0.37 | 1.03 | 2 | 102 | 39025 | 1.20 | 0.45 |
| AT3G08940.1 | LHCB4.2 light harvesting complex PS II | 1.71 | 0.37 | 1.08 | 2 | 222 | 25157 | 1.22 | 0.44 |
| AT3G08940.2 | LHCB4.2 light harvesting complex PS II | 1.73 | 0.37 | 1.45 | 4 | 103 | 31539 | 1.23 | 0.44 |
| AT3G26070.1 | Plastid-lipid associated protein PAP / fibrillin family protein | 1.73 | 0.37 | 1.05 | 3 | 216 | 27147 | 1.23 | 0.44 |
| AT2G34430.1 | LHB1B1, LHCB1.4 light-harvest chl-prot complex II subunit B1 | 1.75 | 0.36 | 1.10 | 3 | 145 | 28209 | 1.24 | 0.44 |
| AT5G67030.1 | ABA1, LOS6, NPQ2, ATABA1, ZEP, zeaxanthin epoxidase (ZEP) | 1.77 | 0.36 | 1.11 | 2 | 247 | 74366 | 1.26 | 0.43 |
| AT4G29010.1 | AIM1 Enoyl-CoA hydratase/isomerase family | 1.77 | 0.36 | 1.16 | 3 | 220 | 78208 | 1.26 | 0.43 |
| ATCG00130.1 | ATPF ATPase, F0 complex, subunit B/B', bacterial/chloroplast | 1.86 | 0.35 | 1.15 | 2 | 108 | 21101 | 1.32 | 0.42 |
| AT5G53490.2 | etratricopeptide repeat (TPR)-like superfamily protein | 1.91 | 0.34 | 1.05 | 2 | 218 | 26013 | 1.35 | 0.41 |
| AT3G63540.1 | Mog1/PsbP/DUF1795-like PS II reaction center PsbP family protein | 1.98 | 0.34 | 1.29 | 2 | 138 | 16326 | 1.40 | 0.40 |
| AT1G55480.1 | ZKT protein containing PDZ domain, a K-box domain, and a TPR region | 1.99 | 0.33 | 1.12 | 2 | 152 | 38016 | 1.41 | 0.40 |
| AT1G29930.1 | CAB1, AB140, CAB140, LHCB1.3 chlorophyll A/B binding protein 1 | 2.03 | 0.33 | 1.16 | 5 | 179 | 28280 | 1.44 | 0.40 |
| AT1G50450.1 | Saccharopine dehydrogenase | 2.11 | 0.32 | 1.37 | 2 | 121 | 46758 | 1.49 | 0.39 |
| ATCG00350.1 | PSAA PS I, PsaA/PsaB protein | 2.16 | 0.32 | 1.13 | 4 | 137 | 83406 | 1.53 | 0.38 |
| AT2G34420.1 | LHB1B2, LHCB1.5 PS II light harvesting complex gene B1B2 | 2.20 | 0.31 | 1.09 | 3 | 158 | 28093 | 1.56 | 0.38 |
| AT5G35170.1 | Adenylate kinase family protein | 2.26 | 0.31 | 1.08 | 2 | 211 | 66039 | 1.60 | 0.37 |
| AT3G01480.1 | CYP38, ATCYP38 cyclophilin 38 | 2.29 | 0.30 | 1.08 | 2 | 331 | 48180 | 1.62 | 0.37 |
| ATCG00900.1 | RPS7.1, RPS7 Ribosomal protein S7p/S5e family protein | 2.40 | 0.29 | 1.04 | 3 | 145 | 17347 | 1.70 | 0.35 |
| AT5G46110.1 | APE2, TPT Glucose-6-phosphate/phosphate translocator-related | 2.45 | 0.29 | 1.00 | 2 | 117 | 44775 | 1.74 | 0.35 |
| AT1G29910.1 | CAB3, AB180, LHCB1.2 chlorophyll A/B binding protein 3 | 2.49 | 0.29 | 1.09 | 4 | 162 | 28266 | 1.76 | 0.34 |
| AT4G10340.1 | LHCB5 light harvesting complex of PS II 5 | 2.50 | 0.29 | 1.16 | 3 | 148 | 30195 | 1.78 | 0.34 |
| AT3G15190.1 | Cloroplast 30S ribosomal protein S20, putative c | 2.51 | 0.28 | 1.07 | 3 | 430 | 22098 | 1.78 | 0.34 |
| AT3G63490.1 | Ribosomal protein L1p/L10e family | 2.54 | 0.28 | 1.04 | 5 | 420 | 37780 | 1.80 | 0.34 |
| AT3G44890.1 | RPL9 ribosomal protein L9 | 2.54 | 0.28 | 1.03 | 4 | 179 | 22177 | 1.80 | 0.34 |
| AT5G66190.1 | ATLFNR1, FNR1 ferredoxin-NADP(+)-oxidoreductase 1 | 2.57 | 0.28 | 2.94 | 3 | 162 | 40643 | 1.82 | 0.34 |
| AT5G19940.1 | Plastid-lipid associated protein PAP / fibrillin family protein | 2.67 | 0.27 | 1.14 | 6 | 324 | 26580 | 1.89 | 0.33 |
| AT1G15820.1 | LHCB6, CP24 light harvesting complex PS II subunit 6 | 2.73 | 0.27 | 1.02 | 3 | 106 | 27505 | 1.94 | 0.32 |
| AT3G27830.1 | RPL12-A, RPL12 ribosomal protein L12-A | 2.77 | 0.27 | 1.01 | 3 | 522 | 20063 | 1.97 | 0.32 |
| AT3G25920.1 | RPL15 ribosomal protein L15 | 3.13 | 0.24 | 1.08 | 2 | 241 | 29860 | 2.22 | 0.29 |
| AT5G54600.1 | Translation protein SH3-like family protein | 3.18 | 0.24 | 1.10 | 3 | 229 | 22077 | 2.26 | 0.29 |
| AT5G57030.1 | LUT2 Lycopene beta/epsilon cyclase protein | 3.33 | 0.23 | 5.26 | 2 | 229 | 59081 | 2.36 | 0.28 |
| ATCG00830.1 | RPL2.1 ribosomal protein L2 | 3.45 | 0.22 | 1.12 | 2 | 184 | 30131 | 2.45 | 0.27 |
| AT1G05190.1 | emb2394 Ribosomal protein L6 family | 3.64 | 0.22 | 1.20 | 3 | 240 | 24747 | 2.59 | 0.26 |
| AT2G05070.1 | LHCB2.2, LHCB2 PS II light harvesting complex gene 2.2 | 4.84 | 0.17 | 3.98 | 3 | 126 | 28659 | 3.43 | 0.21 |
| AT3G25760.1 | AOC1, ERD12 allene oxide cyclase 1 | 5.41 | 0.16 | 1.46 | 2 | 316 | 27841 | 3.84 | 0.19 |
