# Supplementary material for: The chloroplast‐localized small heat shock protein Hsp21 associates with the thylakoid membranes in heat‐stressed plants
Source: Protein Sci. 2017 Jun 26;26(9):1773–84. doi: 10.1002/pro.3213 (PMC5563132; doi:10.1002/pro.3213)
Supplement: Supplementary file 5 — Supporting Information Table 2. [file PRO-26-1773-s005.docx]

**Table S2. Quantification of the relative amount of proteins in the chloroplast soluble stroma from heat-stressed compared to control plants.** Proteins were detected in a 1:1 mixture of soluble stroma fraction from heat-stressed ^15^N-plants and control ^14^N-plants and the L/H ratio determined as outlined in Fig. S1. Protein identification was obtained for 75 proteins with score > 100 and determined L/H ratio with SD_geo_ >1 in Mascot Distiller Quantitation toolbox. Based on the assumption made in Table 3 that the membrane protein subunits in the chlorophyll-protein complex of Photosystem 2 do not change in abundance during 2 h heat stress the determined L/H-ratios and %H-values are multiplied by a factor 0.71 and 1.2, respectively, to reflect a situation with no changes in the 15 proteins. Mass spectrometry raw data related are made available publically at ProteomeXchange (http://www.proteomexchange.org/).

Samples from approximately 20 excised bands were pooled into only 6 runs with LC-MSMS, otherwise processing was performed as described for the thylakoid membrane fractions in Table S1.

| **Accession** | **Description** | **L/H** | **%H** | **SD(geo)** | **# pept** | **Score** | **L/H*0.71** | **%H*1.2** |
| --- | --- | --- | --- | --- | --- | --- | --- | --- |
|  |  |  |  |  |  |  |  |  |
| AT4G24280.1 | cpHsc70-1 chloroplast heat shock protein 70-1 | 0.32 | 0.76 | 1.08 | 6 | 252 | 0.23 | 0.91 |
| ATCG00490.1 | RBCL ribulose-bisphosphate carboxylases | 0.81 | 0.55 | 1.08 | 17 | 1465 | 0.57 | 0.66 |
| AT5G35630.1 | GS2, GLN2, ATGSL1 glutamine synthetase 2 | 0.85 | 0.54 | 1.17 | 4 | 172 | 0.60 | 0.65 |
| AT5G17920.1 | ATCIMS, ATMETS, ATMS1 Cobalamin-independent synthase | 0.87 | 0.53 | 1.10 | 3 | 193 | 0.62 | 0.64 |
| AT2G28000.1 | CPN60A, CH-CPN60A, SLP chaperonin-60alpha | 0.89 | 0.53 | 1.05 | 9 | 992 | 0.64 | 0.63 |
| AT1G20620.1 | CAT3, SEN2, ATCAT3 catalase 3 | 0.91 | 0.52 | 1.01 | 3 | 212 | 0.65 | 0.63 |
| AT3G12780.1 | PGK1 phosphoglycerate kinase 1 | 0.98 | 0.50 | 1.06 | 7 | 722 | 0.70 | 0.60 |
| AT1G23740.1 | Oxidoreductase, zinc-binding dehydrogenase family protein | 1.01 | 0.50 | 1.08 | 4 | 233 | 0.72 | 0.60 |
| AT1G67090.1 | RBCS1A ribulose bisphosphate carboxylase small chain 1A | 1.03 | 0.49 | 1.02 | 3 | 102 | 0.73 | 0.59 |
| AT2G21170.1 | TIM, PDTPI triosephosphate isomerase | 1.04 | 0.49 | 1.06 | 4 | 271 | 0.74 | 0.59 |
| ATCG00490.1 | RBCL ribulose-bisphosphate carboxylases | 1.04 | 0.49 | 1.13 | 8 | 469 | 0.74 | 0.59 |
| AT1G55490.1 | CPN60B, LEN1 chaperonin 60 beta | 1.04 | 0.49 | 1.08 | 5 | 667 | 0.74 | 0.59 |
| AT3G55800.1 | SBPASE sedoheptulose-bisphosphatase | 1.04 | 0.49 | 1.09 | 9 | 423 | 0.74 | 0.59 |
| AT5G04140.1 | GLU1, GLS1, GLUS, FD-GOGAT glutamate synthase 1 | 1.07 | 0.48 | 1.23 | 4 | 464 | 0.76 | 0.58 |
| AT5G38410.1 | Ribulose bisphosphate carboxylase (small chain) family protein | 1.14 | 0.47 | 1.11 | 3 | 116 | 0.81 | 0.56 |
| AT5G36700.1 | ATPGLP1, PGLP1 2-phosphoglycolate phosphatase 1 | 1.14 | 0.47 | 1.13 | 5 | 130 | 0.81 | 0.56 |
| ATCG00490.1 | RBCL ribulose-bisphosphate carboxylases | 1.19 | 0.46 | 1.07 | 7 | 775 | 0.84 | 0.55 |
| AT1G16880.1 | uridylyltransferase-related | 1.19 | 0.46 | 1.06 | 3 | 128 | 0.84 | 0.55 |
| ATCG00490.1 | RBCL ribulose-bisphosphate carboxylases | 1.22 | 0.45 | 1.08 | 11 | 887 | 0.87 | 0.54 |
| AT1G07320.1 | RPL4 ribosomal protein L4 | 1.24 | 0.45 | 1.02 | 3 | 150 | 0.88 | 0.54 |
| AT5G38410.1 | Ribulose bisphosphate carboxylase (small chain) family protein | 1.30 | 0.43 | 1.09 | 5 | 130 | 0.92 | 0.52 |
| AT3G27850.1 | RPL12-C ribosomal protein L12-C | 1.33 | 0.43 | 1.09 | 4 | 314 | 0.94 | 0.52 |
| AT2G04039.2 | unknown protein; FUNCTIONS IN: molecular_function unknown | 1.36 | 0.42 | 1.05 | 3 | 126 | 0.97 | 0.51 |
| AT4G38970.1 | FBA2 fructose-bisphosphate aldolase | 1.41 | 0.42 | 1.18 | 8 | 355 | 1.00 | 0.50 |
| AT1G20620.1 | CAT3, SEN2, ATCAT3 catalase 3 | 1.46 | 0.41 | 1.05 | 3 | 351 | 1.04 | 0.49 |
| AT2G37660.1 | NAD(P)-binding Rossmann-fold superfamily protein | 1.47 | 0.41 | 1.09 | 3 | 151 | 1.04 | 0.49 |
| AT5G26000.2 | TGG1, BGLU38 thioglucoside glucohydrolase 1 | 1.47 | 0.40 | 1.03 | 3 | 207 | 1.04 | 0.49 |
| AT5G38420.1 | Ribulose bisphosphate carboxylase (small chain) family protein | 1.47 | 0.40 | 1.07 | 5 | 105 | 1.05 | 0.49 |
| AT5G25980.2 | TGG2, BGLU37 glucoside glucohydrolase 2 | 1.50 | 0.40 | 1.10 | 3 | 119 | 1.06 | 0.48 |
| AT5G27850.1 | Ribosomal protein L18e/L15 superfamily protein | 1.50 | 0.40 | 1.20 | 3 | 225 | 1.07 | 0.48 |
| AT3G60750.1 | Transketolase | 1.51 | 0.40 | 1.17 | 9 | 643 | 1.07 | 0.48 |
| AT4G21280.1 | PSBQ, PSBQA, PSBQ-1 photosystem II subunit QA | 1.53 | 0.40 | 1.11 | 6 | 627 | 1.09 | 0.47 |
| AT5G01530.1 | LHCB4.1 light harvesting complex photosystem II | 1.53 | 0.39 | 1.14 | 5 | 453 | 1.09 | 0.47 |
| AT3G08940.2 | LHCB4.2 light harvesting complex photosystem II | 1.55 | 0.39 | 1.12 | 5 | 392 | 1.10 | 0.47 |
| AT4G04640.1 | ATPC1 ATPase, F1 complex, gamma subunit protein | 1.56 | 0.39 | 1.11 | 6 | 418 | 1.11 | 0.47 |
| AT2G21330.1 | FBA1 fructose-bisphosphate aldolase 1 | 1.58 | 0.39 | 1.27 | 8 | 406 | 1.12 | 0.47 |
| ATCG00680.1 | PSBB photosystem II reaction center protein B | 1.58 | 0.39 | 1.05 | 3 | 129 | 1.12 | 0.46 |
| AT1G15820.1 | LHCB6, CP24 light harvesting complex photosystem II subunit 6 | 1.60 | 0.39 | 1.06 | 6 | 492 | 1.13 | 0.46 |
| AT1G32990.1 | PRPL11 plastid ribosomal protein l11 | 1.60 | 0.38 | 1.02 | 3 | 208 | 1.14 | 0.46 |
| AT2G05070.1 | LHCB2.2, LHCB2 photosystem II light harvesting complex gene 2.2 | 1.61 | 0.38 | 1.46 | 4 | 123 | 1.14 | 0.46 |
| ATCG00900.1 | RPS7.1, RPS7 Ribosomal protein S7p/S5e family protein | 1.62 | 0.38 | 1.04 | 5 | 398 | 1.15 | 0.46 |
| ATCG00480.1 | ATPB, PB ATP synthase subunit beta | 1.64 | 0.38 | 1.08 | 7 | 607 | 1.16 | 0.45 |
| ATCG00340.1 | PSAB Photosystem I, PsaA/PsaB protein | 1.64 | 0.38 | 1.11 | 3 | 280 | 1.16 | 0.45 |
| ATCG00350.1 | PSAA Photosystem I, PsaA/PsaB protein | 1.65 | 0.38 | 1.10 | 5 | 164 | 1.17 | 0.45 |
| AT2G34420.1 | LHB1B2, LHCB1.5 photosystem II light harvesting complex gene B1B2 | 1.66 | 0.38 | 1.18 | 3 | 124 | 1.18 | 0.45 |
| AT4G38970.1 | FBA2 fructose-bisphosphate aldolase 2 | 1.67 | 0.38 | 1.09 | 3 | 216 | 1.18 | 0.45 |
| ATCG00480.1 | ATPB, PB ATP synthase subunit beta | 1.67 | 0.37 | 1.08 | 3 | 509 | 1.18 | 0.45 |
| ATCG00280.1 | PSBC photosystem II reaction center protein C | 1.68 | 0.37 | 1.34 | 3 | 354 | 1.19 | 0.45 |
| AT2G17360.1 | Ribosomal protein S4 (RPS4A) family protein | 1.73 | 0.37 | 1.20 | 3 | 156 | 1.23 | 0.44 |
| AT4G24770.1 | RBP31, ATRBP31, CP31, ATRBP33 31-kDa RNA binding protein | 1.73 | 0.37 | 1.08 | 4 | 153 | 1.23 | 0.44 |
| AT5G66570.1 | PSBO-1, OEE1, OEE33, OE33, PS II oxygen-evolving complex 1 | 1.75 | 0.36 | 1.13 | 9 | 397 | 1.25 | 0.44 |
| AT4G09650.1 | ATPD ATP synthase delta-subunit gene | 1.76 | 0.36 | 1.03 | 3 | 323 | 1.25 | 0.44 |
| AT2G34420.1 | LHB1B2, LHCB1.5 photosystem II light harvesting complex gene B1B2 | 1.77 | 0.36 | 1.25 | 4 | 213 | 1.25 | 0.43 |
| ATCG00120.1 | ATPA ATP synthase subunit alpha | 1.78 | 0.36 | 1.15 | 11 | 935 | 1.26 | 0.43 |
| ATCG00480.1 | ATPB, PB ATP synthase subunit beta | 1.83 | 0.35 | 1.13 | 11 | 891 | 1.30 | 0.42 |
| AT2G21330.1 | FBA1 fructose-bisphosphate aldolase 1 | 1.85 | 0.35 | 1.14 | 5 | 394 | 1.32 | 0.42 |
| ATCG00540.1 | PETA photosynthetic electron transfer A | 1.86 | 0.35 | 1.12 | 4 | 368 | 1.32 | 0.42 |
| AT1G72370.1 | P40, AP40, RP40, RPSAA 40s ribosomal protein SA | 1.93 | 0.34 | 1.05 | 3 | 142 | 1.37 | 0.41 |
| AT3G60750.1 | Transketolase | 1.95 | 0.34 | 1.13 | 3 | 397 | 1.39 | 0.41 |
| AT3G50820.1 | PSBO2, PSBO-2, OEC33 photosystem II subunit O-2 | 1.98 | 0.34 | 1.08 | 16 | 918 | 1.40 | 0.40 |
| AT1G20020.1 | ATLFNR2, FNR2 ferredoxin-NADP(+)-oxidoreductase 2 | 1.99 | 0.33 | 1.05 | 4 | 277 | 1.41 | 0.40 |
| AT5G66570.1 | PSBO-1, OEE1, OEE33, OE33, PS II oxygen-evolving complex 1 | 2.00 | 0.33 | 1.08 | 15 | 957 | 1.42 | 0.40 |
| ATCG00490.1 | RBCL ribulose-bisphosphate carboxylases | 2.01 | 0.33 | 3.64 | 7 | 236 | 1.42 | 0.40 |
| AT3G26650.1 | GAPA, GAPA-1 glyceraldehyde 3-phosphate dehydrogenase A subunit | 2.17 | 0.32 | 1.12 | 3 | 206 | 1.54 | 0.38 |
| AT1G29910.1 | CAB3, AB180, LHCB1.2 chlorophyll A/B binding protein 3 | 2.34 | 0.30 | 1.93 | 5 | 209 | 1.66 | 0.36 |
| AT1G06680.1 | PSBP-1, OEE2, PSII-P, OE23 photosystem II subunit P-1 | 2.35 | 0.30 | 1.16 | 4 | 219 | 1.67 | 0.36 |
| AT5G66190.1 | ATLFNR1, FNR1 ferredoxin-NADP(+)-oxidoreductase 1 | 2.37 | 0.30 | 1.15 | 5 | 347 | 1.68 | 0.36 |
| ATCG00480.1 | ATPB, PB ATP synthase subunit beta | 2.56 | 0.28 | 1.05 | 3 | 361 | 1.82 | 0.34 |
| AT4G10340.1 | LHCB5 light harvesting complex of photosystem II 5 | 2.56 | 0.28 | 2.05 | 3 | 189 | 1.82 | 0.34 |
| AT3G23400.1 | FIB4 Plastid-lipid associated protein PAP / fibrillin family protein | 2.60 | 0.28 | 1.02 | 3 | 311 | 1.85 | 0.33 |
| AT1G29910.1 | CAB3, AB180, LHCB1.2 chlorophyll A/B binding protein 3 | 2.65 | 0.27 | 2.57 | 4 | 140 | 1.88 | 0.33 |
| ATCG00120.1 | ATPA ATP synthase subunit alpha | 3.16 | 0.24 | 2.82 | 5 | 469 | 2.24 | 0.29 |
| AT2G05070.1 | LHCB2.2, LHCB2 photosystem II light harvesting complex gene 2.2 | 3.18 | 0.24 | 2.88 | 3 | 108 | 2.26 | 0.29 |
| AT4G05180.1 | PSBQ, PSBQ-2, PSII-Q photosystem II subunit Q-2 | 3.38 | 0.23 | 1.15 | 3 | 380 | 2.40 | 0.27 |
| AT5G23120.1 | HCF136 photosystem II stability/assembly factor, chloroplast | 3.91 | 0.20 | 1.12 | 4 | 409 | 2.78 | 0.24 |
| AT3G45140.1 | LOX2, ATLOX2 lipoxygenase 2 | 7.74 | 0.11 | 1.04 | 4 | 513 | 5.50 | 0.14 |
